# Timing of glucocorticoid administration: a cross-sectional survey of glucocorticoid users in an online social network for health

**DOI:** 10.1093/rheumatology/kew421

**Published:** 2016-12-10

**Authors:** Ruth Costello, Rikesh Patel, Jennifer Humphreys, John McBeth, William G. Dixon

**Affiliations:** 1 Arthritis Research UK Centre for Epidemiology, Centre for Musculoskeletal Research, School of Biological Sciences; 2 Health eResearch Centre, Manchester Academic Health Science Centre, University of Manchester; 3 NIHR Manchester Musculoskeletal Biomedical Research Unit, Central Manchester University Hospitals NHS Foundation Trust, Manchester Academic Health Science Centre, Manchester, UK

Rheumatology key message

- The timing of glucocorticoid administration varies significantly in patients, potentially influencing effectiveness and side effects.

Sir, there is a growing body of evidence that the timing of glucocorticoid (GC) administration may be important in reducing symptoms of RA, with evening or night-time doses being shown to reduce morning stiffness [1–3]. A modified-release prednisolone, which is taken in the evening and releases prednisolone 4 h later to coincide with the body’s circadian rhythm of naturally occurring cortisol, has been shown to reduce morning stiffness compared with standard prednisolone taken in the morning [4] and placebo [5]. There is emerging evidence that steroid receptors are differentially expressed in different organs at different times of the day [6], therefore the timing of treatment may affect side-effect profiles. However, it is often recommended that GCs should be taken in the morning due to side effects such as adrenal suppression [7]. It is not known what time patients actually take GCs, therefore the aim of this study was to determine the time people take their GCs.

A short survey of GC users was conducted through Healthunlocked.com, an online social network for health. When users visited a post with the title word 'steroid' or the tags 'glucocorticoid', 'prednisolone', 'prednisone', 'steroid' or 'dexamethasone' the survey popped up for completion. The survey started with a screening question to determine whether respondents were current GC users or had used GCs in the last month. If so, the survey continued with six further questions about the respondent’s age, gender, GC use and the timing of GC administration. The survey was live for 3 months. During the survey, respondents were asked, ‘Do you take your daily dose of steroids once per day, or do you split the dose over two or more times through the day?’ Followed by ‘What time do you normally take your steroid tablets?’ Respondents could indicate the time(s) using a 24 h clock. The study received ethics approval from the University of Manchester Research Ethics Committee (reference 15496). As respondents did not provide identifiable information, informed consent was not required.

At the end of the 3 month survey period 637 respondents had answered the dose and timing questions. Of those, 598 (93.9%) had one dose per day and 39 (6.1%) had two or more doses per day. The majority [*n* = 557 (93.1%)] of single-dose respondents had their dose in the morning, though there was variation within this. A total of 145 (24.2%) respondents usually took their dose between 6 and 7.59 a.m., 320 (54%) between 8 and 9.59 a.m. and 62 (10%) between 10 and 11.59 a.m. (Table 1).

**Table 1:** Time of GC administration by the number of doses per day (*n* = 637)

Time	Single dose, *n* (%)	Two doses per day, *n* (%)		Three doses per day, *n* (%)		
		Dose 1	Dose 2	Dose 1	Dose 2	Dose 3
12–5.59 a.m.	18 (3)	2 (6.5)	–	–	2 (25)	1 (12.5)
6–7.59 a.m.	145 (24.3)	8 (25.8)	–	2 (25)	–	–
8–9.59 a.m.	320 (53.5)	16 (51.6)	1 (3.2)	6 (75)	–	–
10–11.59 a.m.	62 (10.4)	3 (9.7)	1 (3.2)	–	1 (12.5)	–
12–1.59 p.m.	12 (2)	1 (3.2)	1 (3.2)	–	4 (50)	1 (12.5)
2–3.59 p.m.	–	–	2 (6.5)	–	–	–
4–5.59 p.m.	4 (0.7)	–	3 (9.7)	–	1 (12.5)	1 (12.5)
6–8.59 p.m.	16 (2.7)	1 (3.2)	14 (45.2)	–	–	5 (62.5)
9–11.59 p.m.	21 (3.5)	–	9 (29)	–	–	–

Similarly, for those who indicated having multiple doses, there were patterns in the times GCs were taken, but still variation within these patterns. For example, of those taking two doses, the majority had their first dose in the morning and the second in the afternoon, but the time of the first and second dose ranged from 12 a.m. to 8.59 p.m. and 8 a.m. to 11.59 p.m., respectively (Table 1).

To the best of our knowledge, this is the first study reporting the times people take GCs. The results show that although many people take GCs in the morning, there is still variation within this. The evidence suggests this could be important in terms of the effectiveness of GCs and the side effects people may experience and may provide an opportunity to improve outcomes.

*Funding*: This work was supported by the Arthritis Research UK Centre for Epidemiology (20380).

*Disclosure statement:* The authors have declared no conflicts of interest.
